# Abnormal eye movements: relationship with clinical symptoms and predictive value for Alzheimer’s disease

**DOI:** 10.3389/fnagi.2024.1471698

**Published:** 2024-11-21

**Authors:** Jing Qi, Tenghong Lian, Peng Guo, Mingyue He, Jinghui Li, Jing Li, Dongmei Luo, Yanan Zhang, Yue Huang, Gaifen Liu, Zijing Zheng, Huiying Guan, Weijia Zhang, Hao Yue, Zhan Liu, Fan Zhang, Yao Meng, Ruidan Wang, Wenjing Zhang, Wei Zhang

**Affiliations:** ^1^Department of Neurology, Beijing Tiantan Hospital, Capital Medical University, Beijing, China; ^2^Center for Cognitive Neurology, Department of Neurology, Beijing Tiantan Hospital, Capital Medical University, Beijing, China; ^3^Department of Blood Transfusion, Beijing Tiantan Hospital, Capital Medical University, Beijing, China; ^4^China National Clinical Research Center for Neurological Diseases, Beijing Tiantan Hospital, Capital Medical University, Beijing, China

**Keywords:** Alzheimer’s disease, eye movements, lateral fixation, saccade, clinical symptoms, predictive value

## Abstract

**Background:**

Abnormal eye movements occur at the early stages of Alzheimer’s disease (AD). However, the characteristics of abnormal eye movements of patients with AD and their relationship with clinical symptoms remain inconsistent, and their predictive value for diagnosing and monitoring the progression of AD remains unclear.

**Methods:**

A total of 42 normal controls, 63 patients with mild cognitive impairment due to AD (AD-MCI), and 49 patients with dementia due to AD (AD-D) were recruited. Eye movements were assessed using the EyeKnow eye-tracking and analysis system. Cognitive function, neuropsychiatric symptoms, and activities of daily living were evaluated using various rating scales, and correlation analyses and receiver operating characteristic curves were performed.

**Results:**

Patients with AD exhibited increased number of offsets and offset degrees, prolonged offset duration, and decreased accuracy in lateral fixation; reduced accuracy, prolonged saccadic duration, and decreased velocity in prosaccade; decreased accuracy and corrected rate, prolonged corrected antisaccadic duration, and reduced velocity in antisaccade; and reduced accuracy and increased inhibition failures in memory saccade. Eye movement parameters were correlated with global cognition and the cognitive domains of memory, language, attention, visuospatial ability, execution function, and activities of daily living. Subgroup analysis indicated that the associations between eye movements and clinical symptoms in patients with AD were influenced by disease severity and history of diabetes. In the AD-D and AD with diabetes groups, these associations diminished. Nevertheless, the associations persisted in the AD-MCI and AD without diabetes groups. The areas under the curves for predicting AD, AD-MCI, and AD-D were 0.835, 0.737, and 0.899, respectively (all *p* < 0.05).

**Conclusion:**

Patients with AD exhibit distinct patterns of abnormal eye movements. Abnormal eye movements are significantly correlated with global cognition, multiple cognitive domains, and activities of daily living. Abnormal eye movements have a considerable predictive value for the diagnosis and progression of AD.

## 1 Introduction

Alzheimer’s disease (AD) is the most common cognitive disorder and is characterized by a progressive decline in cognitive function, emergence of neuropsychiatric symptoms, and impairment of activities of daily living (ADL). Early diagnosis and treatment can significantly improve the quality of life of patients with AD. However, the early diagnosis is constrained by invasive cerebrospinal fluid tests and the high cost of positron emission tomography.

Eye movement tests serve as behavioral methods for evaluating cognitive function, providing millisecond-level assessments and quantitative parameters. They are non-invasive, inexpensive, and convenient to use. In contrast to traditional rating scales, eye movement tests are unaffected by language and culture, thereby reducing psychological stress in patients. They have been utilized in neuroscience research, encompassing AD, Parkinson’s disease, frontotemporal dementia, and other neurodegenerative diseases, to assess cognitive function (Anderson and MacAskill, 2013; Sekar et al., 2024). Eye movement tests may provide a viable approach for identifying individuals at a high risk of AD, as they are associated with multiple cognitive, perceptual, and motor processes, including attention, working memory, processing speed, motion processing, and inhibition (Hutton, 2008). Nevertheless, a consensus regarding the characteristics of abnormal eye movements in patients with AD and their association with clinical symptoms remains elusive, and their predictive value for AD diagnosis and progression remains ambiguous.

Patients with AD exhibit a diverse array of eye movement disturbances. The paradigms that have been extensively studied include central fixation, prosaccades, and antisaccades. Conversely, another voluntary eye movement, known as a memory saccade, remains relatively underexplored in AD. In central fixation tests, patients with AD exhibited a higher frequency of oblique microsaccades, and the tiny gaze movements occurring during fixation may be attributed to enhanced visual perception (Kapoula et al., 2014) as well as more frequent, oblique saccadic intrusions, including rescanning back to the target after a sudden off-target gaze (Moser et al., 1995). In prosaccade tests, patients with AD exhibited prolonged latency (Opwonya et al., 2022a; Zhang et al., 2023), reduced accuracy (Zhang et al., 2023) and velocity (Fletcher and Sharpe, 1986), and hypometric saccades (Molitor et al., 2015; Chehrehnegar et al., 2019). In antisaccade tests, patients with AD exhibited prolonged latency (Opwonya et al., 2022a; Zhang et al., 2023; Noiret et al., 2018), reduced accuracy (Opwonya et al., 2022a; Zhang et al., 2023; Chehrehnegar et al., 2022; Wilcockson et al., 2019; Kahana Levy et al., 2018) and corrected rate of antisaccades (Wilcockson et al., 2019; Chehrehnegar et al., 2022; Lage et al., 2020; Zhang et al., 2023), and prolonged reaction time of corrected antisaccades (Lage et al., 2020; Noiret et al., 2018). In memory saccade tests, patients with AD demonstrated lower accuracy, a higher incidence of negative errors, and longer latency than normal controls (NC) (Lage et al., 2020). A study that employed video electronystagmograms to evaluate memory saccades revealed prolonged latency and decreased accuracy in patients with mild cognitive impairment (MCI) due to AD (AD-MCI) (Bai et al., 2010).

However, the conclusions from various studies are inconsistent. Patients with AD had normal prosaccadic function (Molitor et al., 2015), exhibiting comparable prosaccade accuracy, latency, and velocity. No consensus has been reached regarding whether differences exist in latency and accuracy between patients with AD-MCI and those with dementia due to AD (AD-D) (Zhang et al., 2023). Regarding the latency of antisaccades, no significant differences were identified between patients with AD and NC (Opwonya et al., 2022b). Additionally, a meta-analysis demonstrated longer latency in NC than in patients with AD (Kahana Levy et al., 2018). Concerning eye movement paradigms, relatively few studies have employed lateral fixation (Weng et al., 2023). Compared to central fixation, lateral fixation significantly activated the frontal–parietal structures of the eye movement system, including the frontal eye field (FEF), parietal eye field (PEF), dorsal lateral prefrontal cortex (DLPFC), and supplementary motor area (Deutschländer et al., 2005). Consequently, lateral fixation serves as a more effective method for assessing the function of these areas, thereby facilitating the evaluation of neurocognitive functions, such as visual attention and inhibitory control in patients with AD. Additionally, this study placed greater emphasis on gaze deviations attributable to attention deficits than physiological fixation deviations, such as microsaccades and saccadic intrusions, which are frequently used in central fixation. Therefore, the evaluation of fixation stability through lateral fixation is recommended. The existing literature on memory saccades is limited; however, this body of research is hindered by various constraints, including small sample sizes (Lage et al., 2020), simplistic parameter settings (Rane et al., 2023) and patients without AD (Bai et al., 2010). Consequently, the evaluation of eye movement characteristics in patients at different stages of AD using multidimensional parameters in a relatively larger sample size is necessary.

To the best of our knowledge, no investigations of the correlation between lateral fixation parameters and cognitive function of patients with AD exist. Prosaccades were reportedly related to global cognition and executive function in patients with AD (Zhang et al., 2023). The latency, accuracy, and velocity of the prosaccades in patients with AD were associated with global cognition (Molitor et al., 2015). Furthermore, the latency of prosaccades was related to spatial memory and visuospatial function (Lage et al., 2020). The latency, accuracy, and reaction time of corrected antisaccades in patients with mild AD were correlated with global cognition, episodic memory, language, attention, and executive function (Noiret et al., 2018). In patients with AD-MCI, the error rate of antisaccades was associated with global cognition (Holden et al., 2018), language (Zhang et al., 2023), and executive function (Heuer et al., 2013; Holden et al., 2018). Concerning memory saccades, only one study identified a correlation between its accuracy and global cognition and verbal memory in patients with mild AD, although the sample size was limited to just ten cases (Lage et al., 2020). In summary, most studies have focused primarily on assessing global cognition (Ionescu et al., 2023), often overlooking the critical relationship between eye movements and specific cognitive domains. Early identification of cognitive domain impairments aids in locating the lesion, enabling earlier intervention and optimization of therapeutic strategies.

An umbrella review, the review of previously published systematic reviews or meta-analyses, revealed that the area under the curve (AUC) for identifying AD-MCI patients based on the latency of prosaccade and antisaccade was 0.64 and 0.62, respectively. The highest accuracy for identifying patients with AD-MCI was the errors in antisaccade task, which yielded an AUC of 0.79 (Costanzo et al., 2023). Furthermore, a previous study indicated that eye-tracking analysis of the King-Devick test facilitated the early detection of AD. The AUCs for differentiating MCI from mild AD-D groups were 0.727 for total time and 0.745 for errors committed during the King-Devick test (Hannonen et al., 2022). Notably, despite the high sensitivity associated with the antisaccade task, its specificity for diagnosing neurodegenerative diseases may be limited. A single task alone might not function as a reliable diagnostic tool; however, the integration of gaze metrics from multiple tasks could improve classification accuracy (Wolf et al., 2023; He et al., 2024). For instance, a study conducted in a Chinese community cohort revealed that a model incorporating smooth pursuit, prosaccade, and antisaccade features achieved an AUC of 0.926 for identifying patients with cognitive impairment (Lin et al., 2024). The AUC for identifying patients with MCI by utilizing eye movement parameters of the prosaccade and Go/No-go tasks was initially 0.715; however, it increased to 0.752 when combined with demographic data (Opwonya et al., 2023). Recently, a cognitive score derived from gaze data while viewing short movies and images based on eye tracking effectively differentiated MCI from NC (Oyama et al., 2019). Additionally, a novel eye-tracking score, calculated as the percentage of time spent gazing at task movies within the regions of interest, distinguished AD and NC, as well as MCI and NC (Tadokoro et al., 2021). Although the combination of different eye-tracking tasks used to identify AD has received widespread attention from scholars, the results of these studies have been inconsistent. To date, a quantitative combination of multiple saccadic movements and lateral fixation has not been used to identify AD and predict its progression.

We propose the hypothesis that patients with AD exhibit abnormal fixational and saccadic eye movements, which occur at the early stage of AD and exacerbate as disease progresses. These eye movement parameters are associated with impaired cognitive function, particularly in the domains of attention, executive and visuospatial functions; hence, they may indicate early identification and progression prediction of AD. Consequently, this study recruited patients with AD at different stages, evaluated eye movements using multidimensional parameters, and assessed their relationship with clinical symptoms by a variety of rating scales. Considering the severity of AD and potential influence of diabetes on oculomotor function, subgroup analyses were conducted. We investigated the predictive value of eye movements for the diagnosis and progression of AD.

## 2 Methods

### 2.1 Ethics approval and consent to participate

This study received ethical approval from the Review Board of Beijing Tiantan Hospital, Capital Medical University. Written informed consent was obtained from all participants and their caregivers.

### 2.2 Participants

A total of 154 participants were recruited from the Beijing Tiantan Hospital between November 2022 and November 2023. Moreover, 63 patients and 49 patients with AD-MCI and AD-D, respectively, were enrolled according to the National Institute of Aging and Alzheimer’s Association criteria. The core clinical criteria for AD-MCI were as follows: (1) subjective complaints of cognitive decline, or alterations in cognitive function as reported by caregivers or clinicians; (2) impairment(s) in one or more cognitive domains, including episodic memory, executive function, attention, language, and visuospatial skills, with significant impairment in episodic memory; (3) maintenance of independence in ADL; and (4) a Clinical Dementia Rating (CDR) score of 0.5 (Albert et al., 2011). The core clinical criteria for AD-D were as follows: (1) Met the diagnostic criteria for dementia: (i) interfered with the ability to function at usual activities or work; (ii) represented a decline compared to previous levels of functioning and performing; (iii) cognitive impairment was diagnosed through a combination of history-taking from the patient, a knowledgeable informant and an objective cognitive assessment; (iv) the cognitive or behavioral impairment involved a minimum of two of the following domains: a. compromised ability to acquire and remember new information; b. impaired ability to reason and handle complex tasks and poor judgment; c. impaired visuospatial function; d. deficits in language function; e. changes in personality, behavior, or comportment; (2) Had the following characteristics: (i) an insidious onset; (ii) a definitive history of worsening of cognition by report or observation; (iii) the initial and most prominent cognitive deficits were manifested in one of the following categories in medical history and examination: a. amnestic presentation, encompassing impairment in learning and recall of recently learned information. There should be evidence of cognitive disorder in at least one other cognitive domain; b. non-amnestic presentations, including language, visuospatial and executive dysfunctions; and (3) a CDR score of ≥1 was required (McKhann et al., 2011).

The exclusion criteria of AD were as follows: (1) neurological disorders besides AD affecting cognition, including vascular cognitive impairment, Lewy body disease, Parkinson’s disease, and frontotemporal degeneration, etc.; (2) systemic diseases, including uncontrolled hypertension, severe chronic disease, etc.; and (3) a history of alcoholism or carbon monoxide poisoning.

The inclusion criteria for NC were as follows: (1) absence of cognitive impairment complaint; (2) Mini-Mental State Examination (MMSE) scores exceeding 17, 20, and 24 for illiterates, primary and junior high school graduates, and individuals with higher education levels, respectively (Wang and Zhang, 1989); (3) a Montreal Cognitive Assessment (MoCA) score > 26 (Nasreddine et al., 2005); (4) a CDR score of 0; and (5) no history of neurological or psychiatric diseases, uncontrolled systemic diseases, or eye diseases affecting vision and eye movements.

The exclusion criteria for eye movement analyses were as follows: (1) eye diseases or neurological disorders that impair vision or eye movement, including optic nerve disease, oculomotor nerve damage, visual field defects, macular disease, retinal detachment, glaucoma, color blindness, ptosis, and non-physiological nystagmus; (2) inability to comprehend the test content or follow instructions; and (3) failure to pass the calibration test and poor data quality.

### 2.3 Demographic and clinical information

Demographic information, including gender, age, duration, years of education, Apolipoprotein E ε4 (*APOE* ε4) allele status, and body mass index (BMI), and clinical information, including smoking, drinking, hypertension, diabetes history, blood pressure, fasting plasma glucose, and glycated hemoglobin were collected. Patients were classified as having diabetes if they satisfied any of the following criteria: (1) fasting plasma glucose≥7.0 mmol/L; (2) random plasma glucose≥11.1 mmol/L; (3) HbA1c ≥ 6.5% (American Diabetes Association Professional Practice Committee, 2022); and (4) a self-reported history of diabetes or the use of diabetes medication.

### 2.4 Assessments of clinical symptoms of AD

#### 2.4.1 Global cognitive function

Global cognitive function was assessed using MMSE (Cockrell and Folstein, 1988) and MoCA (Nasreddine et al., 2005) scales. MMSE scale, which assesses cognitive domains of memory, orientation, language, attention, and calculation ability, is sensitive to AD-D. Individuals with illiteracy, primary education, and junior high school education or higher were considered to have cognitive impairment if their MMSE scores were < 17, <20, and < 24, respectively. The MoCA scale, which evaluates visuospatial and executive functions, naming, memory, language, attention, abstraction, delayed recall, and orientation abilities, is sensitive to AD-MCI. A score of ≤26 indicated potential cognitive impairment. An additional point was awarded if the individual completed <12 years of education.

#### 2.4.2 Individual cognitive domains

Verbal memory was evaluated using the Auditory Verbal Learning Test (AVLT). The AVLT N1-3, AVLT N4, and AVLT N5 evaluate immediate, short-delayed, and long-delayed recalls, respectively, while AVLT N6 measures logical memory (Guo et al., 2009). Delayed visual memory was assessed using the Rey-Osterrieth Complex Figure Test (RFT). Lower AVLT and RFT-delayed memory scores indicate poorer memory performance (Yan et al., 2006). The language was assessed using the Verbal Fluency Test (VFT) (Lin et al., 2014) and Boston Naming Test (BNT) (Sebaldt et al., 2009), with lower scores indicating worse language function. Attention was assessed using the Symbol Digit Modalities Test (SDMT) (Fellows and Schmitter-Edgecombe, 2019) and the Trail Making Test A (TMT-A) (Wei et al., 2018). Decline in SDMT score and extended completion time on the TMT-A revealed compromised attention function. Visuospatial ability was estimated using RFT-imitation (Yan et al., 2006), with lower score indicating worse visuospatial ability. Executive function was evaluated using the Stroop Color and Word Test C (SCWT-C) (Guo et al., 2007) and the Trail Making Test-B (TMT-B) (Wei et al., 2018), with increased completion time reflecting poor execution function.

#### 2.4.3 Assessment of neuropsychiatric symptoms

Global neuropsychiatric symptoms were rated using the Neuropsychiatric Inventory (NPI) (Cummings et al., 1994), wherein elevated scores corresponded to a greater severity of neuropsychiatric symptoms. Furthermore, agitation was assessed using the Cohen-Mansfield Agitation Inventory (CMAI), with higher score indicating more severe agitation (Koss et al., 1997). Apathy was rated using the Modified Apathy Estimate Scale (MAES), wherein a score > 14 revealed clinically meaningful apathy (Starkstein et al., 1992).

#### 2.4.4 Assessment of ADL

The Activities of Daily Living (ADL) scale encompasses basic and instrumental ADL scales, with higher score reflecting poorer performance in ADL (Katz et al., 1963).

### 2.5 Eye movement evaluation

**Figure 1:**
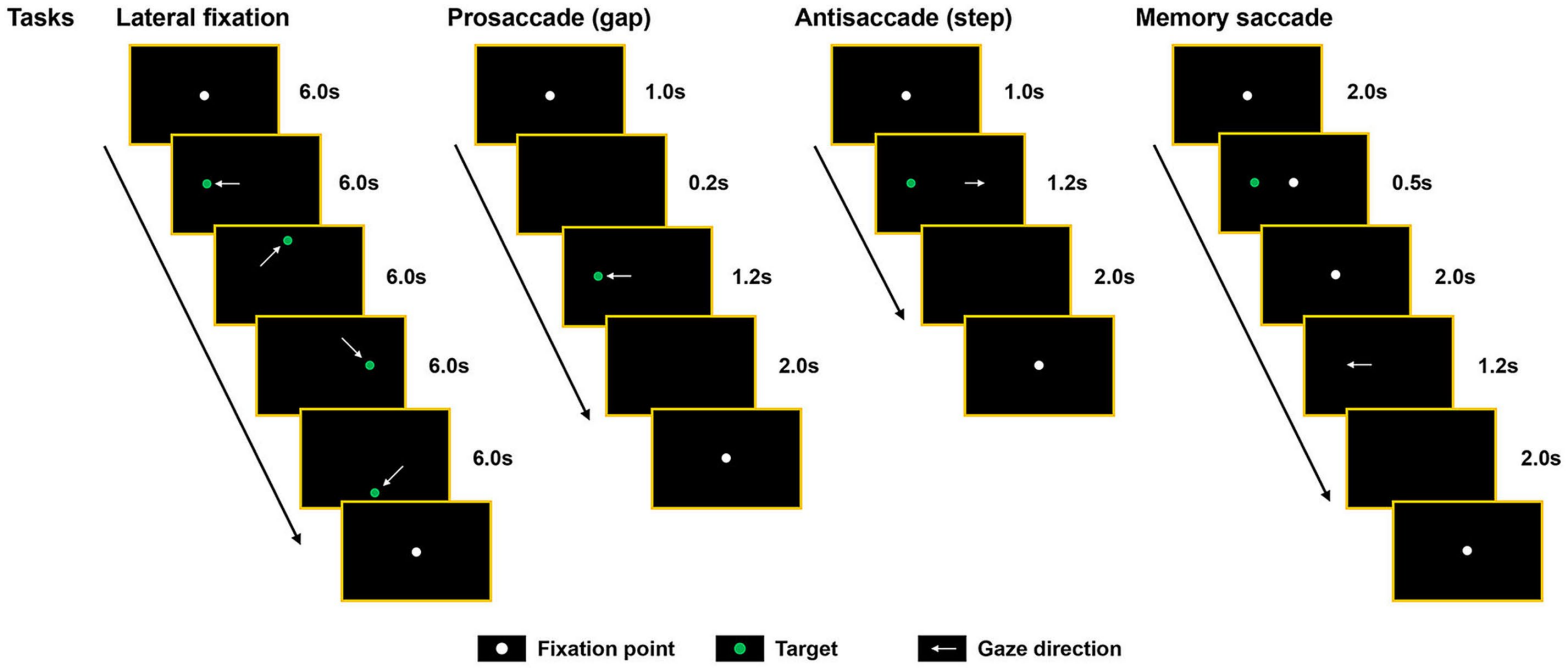
Schematic diagram of the eye movement tasks. Each task commenced with the presentation of a fixation point located at the center of the screen (white dot), necessitating participants to maintain their gaze. The central fixation point either disappeared before (prosaccade) or coincident with (antisaccade and lateral fixation) the appearance of the peripheral target (green dot), or it remained visible on the screen along with the target (memory saccade).

EyeKnow (Beijing CAS-Ruiyi Information Technology Co., Ltd.), an intelligent eye movement analysis and evaluation device based on infrared corneal reflection precise positioning technology, was used to record eye movement data. The apparatus featured a dual-screen display characterized by a refresh rate of 120 Hz, a resolution of 3,664 × 1,920 pixels, a visual angle of 98°, and an infrared acquisition module of 90 Hz. To exclude potential eye diseases, participants underwent an examination by an ophthalmologist. The participants were required to possess either normal or corrected-to-normal vision in at least one eye. The participants were situated in a quiet clinic room, sitting in front of the instrument with the chin resting on a chin rest, and both eyes positioned in front of the acquisition module screen. Before the test, participants were provided with standardized and comprehensive instructions to minimize potential variability. Before the experiment, a nine-point calibration procedure was performed to ensure a maximum calibration error radius of 2°. During the experiment, if the participants needed to take a break or move their heads away from the chin rest for any reason, this step was repeated. Following calibration, the stimuli were displayed on the monitor, and the embedded data processing module analyzed the eye movement parameters. An examination protocol delineating the sequence of tests was followed to minimize variability as much as possible. The sequence of tests included lateral fixation, prosaccade, antisaccade, and memory saccade. A schematic diagram illustrating these tasks is shown in Figure 1. The time required for task completion was approximately 10 min, which encompassed the time allocated for calibration, audiovisual presentation of the task, and task execution.

Eye movement paradigms: (1) Lateral fixation: The target was represented by a green dot, which was displayed in the center or another position. Participants were instructed to fixate on the fixed target as promptly as possible until its disappearance. The target appeared in the center for 6 s with lateral fixation (in the order of left, top, right, and bottom) for 24 s and a deviation angle of 15°. Lateral fixation comprised 20 trials. The number of offset was defined as the total number of fixation points that deviated from the target dot by more than 2° or 4°. The total offset degrees represented the cumulative degree of deviation from the target dot during the task. The total offset duration was defined as the cumulative time at which the fixation point deviated from the target dot by >4°. Accuracy represented the percentage of time allocated for fixation on the target dot (< 4°) relative to the total duration. This study primarily concentrated on gaze deviations attributable to lapses in attention, establishing a threshold of 4° for physiological fixation deviations, such as square-wave jerks, microsaccades, and saccadic intrusions, which typically do not exceed this limit (Anagnostou et al., 2020). Deviations exceeding this threshold are likely attributable to loss of attention. (2) Prosaccade (gap condition): participants were instructed to execute rapid and precise saccades toward the target dot, which was randomly shifted ±15° from the center in both horizontal and vertical directions. The prosaccade was repeated 20 times. Accuracy was defined as the percentage of successful saccades toward the target relative to the total number of trials. Latency represented the interval between the onset of the target dot and the initiation of eye saccades. Mean saccadic duration represented the average time required to execute a correct saccade from the appearance of the target dot in all trials. Velocity was determined by quantifying the angular displacement over the interval spanning from the initiation to the completion of the saccade. (3) Antisaccade (step condition): Participants were instructed to execute a saccade in the direction opposite to that of the target dot immediately following the disappearance of the central point. Following the execution of a non-inhibitory saccade to the target, participants were permitted to attempt a corrective action. Antisaccades comprised 20 trials. Accuracy was defined as the percentage of direct saccades to the opposite direction of the target dot over the total number of trials. Antisaccade duration referred to the interval between the appearance of the target dot and the completion of an accurate antisaccade. The corrected antisaccade rate represented the ratio of the number of corrective saccades executed from the initial point to the opposite position to the number of non-inhibitory saccades, and the corrected antisaccadic duration referred to the time from the onset to the completion of the corrective saccade. The definitions of latency and velocity were analogous to those used in the prosaccade. (4) Memory saccade: At the onset of fixating the central point, participants were instructed to focus on the location of another target dot that would disappear after 0.5 s, situated within a 10° radius of the surroundings. Upon the disappearance of the central point, the participants were required to execute a saccade toward the location where another target dot had previously flickered. The saccade was deemed correct if it landed within a radius of 4 °of the position where the target had disappeared. The number of inhibition failures referred to the number of uninhibited saccades toward the target. Memory deviation was defined as the cumulative degree of deviation from the target dot across all the trials.

### 2.6 Statistical analysis

Statistical analyses were conducted using SPSS (version 26.0; IBM Corp., Armonk, NY, United States). Analysis of variance and the Kruskal-Wallis test were used for normally distributed and non-normally distributed variables, respectively. Post-hoc comparisons were conducted using the Bonferroni and Tamhane’s T2 test when the variance was equal and unequal, respectively. Additionally, the chi-squared test was used to compare categorical variables. Partial Spearman’s correlation was used to calculate the correlation between variables. The models were adjusted for age, sex, duration, years of education, BMI, diastolic blood pressure, *APOE* ε4 allele carriers, and history of diabetes. Subgroup analyses were performed to determine whether disease severity and diabetes influenced the association between eye movement parameters and cognitive function. Independent influencing factors for AD, AD-MCI, and AD-D were identified using multivariate logistic regression and are presented as forest diagrams. Receiver operating characteristic curves and AUCs were used to distinguish patients with NC, AD-MCI, and AD-D. Statistical significance was set at *p* < 0.05.

## 3 Results

### 3.1 Demographic and clinical information

A total of 42 NC, 63 AD-MCI, and 49 AD-D patients were recruited for this study. The AD-D group exhibited a lower BMI and diastolic blood pressure than the NC group. The AD-D group demonstrated a longer disease duration, a higher proportion of *APOE* ε4 allele carriers, and a lower BMI than the AD-MCI group (all *p* < 0.05). The remaining data were comparable among the three groups (Table 1).

**Table 1:** Comparisons of demographic and clinical information among NC, AD-MCI, and AD-D groups.

	NC group (*n* = 42)	AD-MCI group (*n* = 63)	AD-D group (*n* = 49)
Demographic information			
Female [*n* (%)]	21 (50.0%)	35 (55.6%)	33 (67.3%)
Age (years, mean ± SD)	63.48 ± 6.88	65.11 ± 8.43	64.47 ± 11.27
Duration [months, median (Q1-Q3)]	NA	24.00 (12.00, 45.00)	36.00 (24.00, 60.00) ^‡^
Years of education [years, median (Q1-Q3)]	15.00 (12.00, 16.00)	12.00 (9.00, 15.00)	12.00 (9.00, 15.25)
*APOE* ε4 allele carriers [*n* (%)]	NA	10 (16.7%)	29 (60.4%) ^‡‡‡^
BMI (kg/m^2^, mean ± SD)	26.16 ± 3.34	25.09 ± 3.89	22.72 ± 3.18 ^†††, ‡‡^
Clinical information			
Smoking [*n* (%)]	12 (28.6%)	16 (25.4%)	9 (18.4%)
Drinking [*n* (%)]	9 (21.4%)	15 (23.8%)	8 (16.3%)
History			
Hypertension [*n* (%)]	25 (59.5%)	30 (47.6%)	20 (40.8%)
Diabetes mellitus [*n* (%)]	7 (16.7%)	17 (27.0%)	16 (32.7%)
Systolic blood pressure [mmHg, mean ± SD]	135.43 ± 17.24	134.25 ± 17.08	131.08 ± 13.70
Diastolic blood pressure [mmHg, median (Q1-Q3)]	86.00 (76.00, 95.00)	80.00 (74.00, 89.25)	77.00 (74.00, 83.00)^†^
Fasting plasma glucose [mmol/l, median (Q1-Q3)]	5.01 (4.69, 5.55)	5.34 (4.81, 6.15)	5.10 (4.59, 6.38)
Glycated hemoglobin [%, median (Q1-Q3)]	5.12 (4.72, 5.58)	5.31 (4.81, 6.26)	5.09 (4.58, 6.43)

^†p < 0.05 and †††p < 0.001, AD-D versus NC groups; ‡p < 0.05, ‡‡p < 0.01 and ‡‡‡p < 0.001, AD-D versus AD-MCI groups.^

NC, normal control; AD-MCI, mild cognitive impairment due to Alzheimer’s disease; AD-D, dementia due to Alzheimer’s disease; *APOE, apolipoprotein E*; BMI, body mass index; NA, not applicable.

### 3.2 Eye movement parameters

In lateral fixation, the AD-D group demonstrated a greater number of offsets (>4°), larger total offset degrees (>4°), longer total offset duration, and lower accuracy than the NC and AD-MCI groups. The AD-MCI group exhibited a longer total offset duration than the NC group (all *p* < 0.05; Table 2).

**Table 2:** Comparisons of eye movement parameters among NC, AD-MCI, and AD-D groups.

	NC group (*n* = 42)	AD-MCI group (*n* = 63)	AD-D group (*n* = 49)
Lateral fixation			
Number of offset (>4°) [times, median (Q1-Q3)]	13.00 (6.00, 23.00)	16.00 (10.00, 31.00)	51.00 (31.00, 72.00) ^†††, ‡‡^
Number of offset (>2°) [times, median (Q1-Q3)]	40.00 (20.00, 57.00)	49.00 (35.00, 67.00)	49.00 (30.00, 72.00)
Total offset degrees (>4°) [°, median (Q1-Q3)]	113.99 (91.13, 213.86)	143.80 (106.57, 243.00)	305.10 (227.74, 435.37) ^††, ‡^
Total offset duration [ms, median (Q1-Q3)]	2193.31 (1679.70, 3498.20)	2665.30 (2012.78, 6788.19) ^*^	9911.57 (6913.13, 12049.69) ^†††, ‡‡‡^
Accuracy [%, median (Q1-Q3)]	92.67 (88.36, 94.39)	91.10 (77.33, 93.27)	67.02 (59.72, 76.91) ^†††, ‡‡‡^
Prosaccade			
Accuracy [%, median (Q1-Q3)]	100.00 (100.00, 100.00)	100.00 (100.00, 100.00)	100.00 (93.75, 100.00) ^†, ‡^
Latency [ms, median (Q1-Q3)]	238.04 (223.95, 257.00)	229.68 (217.84, 254.64)	239.95 (219.28, 277.40)
Fastest saccadic duration [ms, median (Q1-Q3)]	238.04 (223.95, 257.00)	229.68 (217.84, 254.46)	239.95 (219.28, 277.40) ^†, ‡^
Mean saccadic duration [ms, median (Q1-Q3)]	269.07 (256.51, 300.47)	265.24 (253.40, 312.99)	322.59 (286.84, 420.13) ^†††, ‡‡‡^
Mean saccadic velocity (°/s, mean ± SD)	240.50 ± 54.75	230.03 ± 71.91	185.30 ± 45.58 ^†††^
Maximum saccadic velocity [°/s, median (Q1-Q3)]	473.40 (423.93, 504.65)	481.01 (421.74, 544.21)	423.17 (393.54, 478.05)
Antisaccade			
Accuracy [%, median (Q1-Q3)]	53.33 (31.58, 76.39)	25.00 (7.14, 60.00) ^**^	9.72 (0.00, 25.33) ^†††, ‡‡^
Latency (ms, mean ± SD)	340.99 ± 66.93	318.71 ± 66.30	362.46 ± 91.91
Fastest antisaccadic duration [ms, median (Q1-Q3)]	263.50 (235.78, 305.13)	249.91 (235.23, 290.51)	277.44 (235.59, 304.58)
Mean antisaccadic duration (ms, mean ± SD)	402.14 ± 66.23	381.95 ± 64.79	437.67 ± 116.64
Corrected antisaccade rate [%, median (Q1-Q3)]	100.00 (88.75, 100.00)	92.12 (60.00, 100.00)	13.89 (0.00, 67.86) ^†††, ‡‡‡^
Corrected mean antisaccadic duration [ms, median (Q1-Q3)]	257.03 (204.77, 347.07)	307.24 (245.46, 369.41)	335.44 (166.63, 435.05) ^†††, ‡^
Mean antisaccadic velocity (°/s, mean ± SD)	242.00 ± 62.55	217.74 ± 58.02	200.50 ± 79.73 ^††^
Maximum antisaccadic velocity (°/s, mean ± SD)	507.17 ± 111.38	491.01 ± 137.27	438.77 ± 197.21
Memory saccade			
Accuracy [%, median (Q1-Q3)]	60.00 (40.00, 75.00)	45.00 (10.00, 65.00) ^**^	15.00 (5.00, 30.00) ^†††, ‡‡‡^
Number of inhibition failures [times, median (Q1-Q3)]	4.00 (1.00, 7.00)	5.00 (3.00, 9.00)	10.00 (4.00, 13.00) ^†††, ‡^
Latency [ms, median (Q1-Q3)]	435.95 (377.31, 535.72)	416.06 (353.32, 555.13)	378.51 (334.01, 515.16)
Memory deviation (°, mean ± SD)	3.00 ± 0.84	3.36 ± 1.18	3.82 ± 1.68

^†^*p* < 0.05, ^††^*p* < 0.01 and ^†††^*p* < 0.001, AD-D versus NC groups; ^‡^*p* < 0.05, ^‡‡^*p* < 0.01 and ^‡‡‡^*p* < 0.001, AD-D versus AD-MCI groups. ^*^p < 0.05 and ^**^p < 0.01, AD-MCI versus NC groups.

NC, normal control; AD-MCI, mild cognitive impairment due to Alzheimer’s disease; AD-D, dementia due to Alzheimer’s disease.

In prosaccades, the AD-D group had a lower accuracy, longer fastest and mean saccadic duration than the NC and AD-MCI groups. The AD-D group showed a lower mean saccadic velocity than the NC group (all *p* < 0.05). No significant differences were observed between the NC and AD-MCI groups.

In antisaccade, the AD-D group exhibited lower accuracy, a lower corrected antisaccade rate, and a longer corrected mean antisaccadic duration than the NC and AD-MCI groups. The AD-D group exhibited a lower mean antisaccadic velocity than the NC group (all *p* < 0.05). In addition to the lower accuracy (*p* < 0.01), no differences were observed in the remaining parameters between the AD-MCI and NC groups.

In memory saccades, accuracy ranked from lowest to highest was as follows: AD-D, AD-MCI, and NC groups. The AD-D group experienced a higher number of inhibition failures than the NC and AD-MCI groups (all *p* < 0.05).

**Figure 2:**
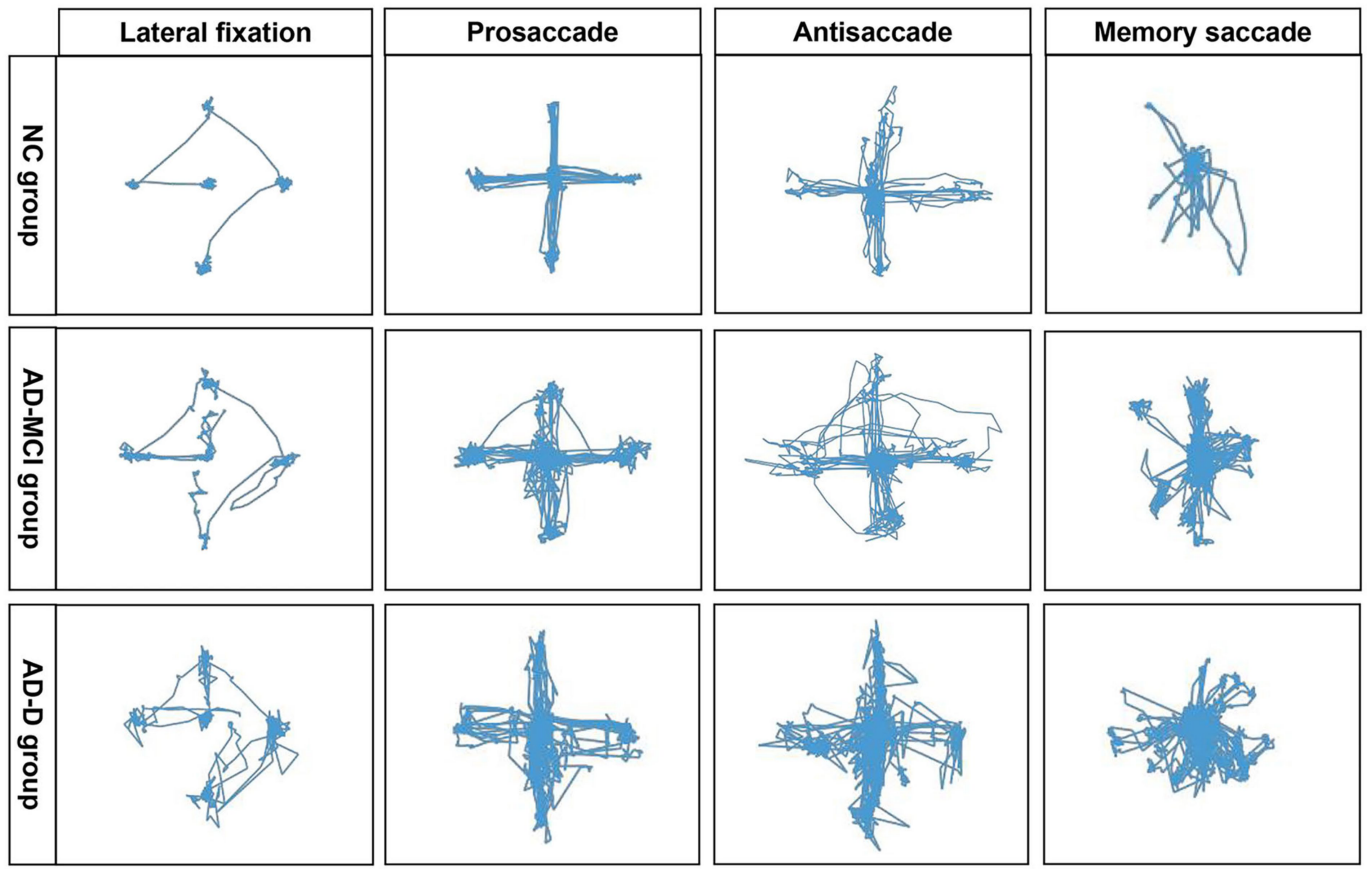
Comparisons of planar gaze trajectories in different eye movement paradigms among NC, AD-MCI, and AD-D groups. NC group exhibited regular and smooth eye movement trajectories; AD-MCI group demonstrated mild trajectory disorder, manifested as unstable and uncoordinated eye movements; AD-D group displayed highly erratic trajectories, with unstable fixations across all tasks. NC, normal control; AD-MCI, mild cognitive impairment due to Alzheimer’s disease; AD-D, dementia due to Alzheimer’s disease.

To visually illustrate the differences, typical eye movement trajectories during the tasks derived for each group are described (Figure 2). The NC group presented a regular and smooth eye movement trajectory, whereas the AD-MCI group displayed a mildly disturbed trajectory, indicating unstable and uncoordinated eye movement. In contrast, the AD-D group exhibited a highly erratic trajectory with unstable fixations across all tasks.

### 3.3 Comparisons of clinical symptoms among NC, AD-MCI, and AD-D groups

Significant differences were identified in global cognition, as assessed by the MMSE and MoCA scales, along with various cognitive domains evaluated by the AVLT, RFT delayed recall, VFT-T, BNT, SDMT, TMT-A, RFT imitation, and TMT-B, revealing the poorest performance in the AD-D group and the highest performance in the NC group (all *p* < 0.05). In addition, the AD-D group had higher scores on the Neuropsychiatric Inventory (NPI), Cohen-Mansfield Agitation Inventory, Modified Apathy Estimate Scale, and ADL scales than the NC and AD-MCI groups (all *p* < 0.01). The AD-MCI group displayed a higher NPI score than the NC group (*p* < 0.05; Table 3).

**Table 3:** Comparisons of clinical symptoms among NC, AD-MCI, and AD-D groups.

	NC group (*n* = 42)	AD-MCI group (*n* = 63)	AD-D group (*n* = 49)
Cognitive function			
Global cognitive function			
MMSE [points, median (Q1-Q3)]	29.00 (28.00, 30.00)	26.00 (21.00, 28.00) ^***^	14.00 (10.00, 17.00) ^†††, ‡‡‡^
MoCA [points, median (Q1-Q3)]	26.00 (25.00, 27.00)	21.00 (15.00, 23.00) ^***^	10.00 (7.00, 12.00) ^†††, ‡‡‡^
Cognitive domains			
Memory			
AVLT N1-3 (points, mean ± SD)	19.79 ± 0.71	15.28 ± 0.64 ^***^	8.24 ± 0.52 ^†††, ‡‡‡^
AVLT N4 [points, median (Q1-Q3)]	6.00 (4.00, 8.00)	3.00 (1.25, 5.00) ^**^	0.00 (0.00, 0.00) ^†††, ‡‡‡^
AVLT N5 [points, median (Q1-Q3)]	6.00 (4.00, 7.00)	3.00 (1.00, 5.75) ^**^	0.00 (0.00, 0.00) ^†††, ‡‡‡^
AVLT N6 [points, median (Q1-Q3)]	6.00 (5.00, 8.00)	3.00 (1.00, 5.00) ^**^	0.00 (0.00, 0.00) ^†††, ‡‡‡^
RFT delayed recall [points, median (Q1-Q3)]	16.50 (10.00, 22.50)	8.00 (1.00, 18.00) ^**^	0.00 (0.00, 0.00) ^†††, ‡‡‡^
Language			
VFT-T [points, median (Q1-Q3)]	53.00 (45.00, 60.75)	45.00 (32.00, 52.00) ^**^	18.00 (13.50, 28.00) ^†††, ‡‡‡^
BNT [points, median (Q1-Q3)]	29.00 (27.00, 29.25)	27.00 (23.00, 28.00) ^**^	21.00 (16.00, 26.00) ^†††, ‡‡‡^
Attention			
SDMT [points, median (Q1-Q3)]	46.00 (39.75, 50.00)	33.00 (23.00, 49.00) ^*^	7.00 (0.00, 18.00) ^†††, ‡‡‡^
TMT-A [seconds, median (Q1-Q3)]	47.00 (37.78, 63.00)	74.00 (49.00, 100.51) ^**^	142.00 (84.74, 240.00) ^†††, ‡‡‡^
Visuospatial ability			
RFT imitation [points, median (Q1-Q3)]	34.00 (32.00, 35.25)	31.00 (23.50, 34.00) ^**^	12.00 (1.50, 26.25) ^†††, ‡‡‡^
Executive function			
SCWT-C [seconds, median (Q1-Q3)]	68.50 (55.25, 79.25)	75.02 (62.00, 96.00)	140.00 (91.88, 196.50) ^†††, ‡‡^
TMT-B [seconds, median (Q1-Q3)]	130.50 (116.75, 158.50)	192.88 (133.00, 240.00) ^**^	240.00 (240.00, 240.00) ^†††, ‡‡‡^
Neuropsychiatry symptoms			
NPI [points, median (Q1-Q3)]	0.00 (0.00, 0.00)	0.00 (0.00, 3.00) ^*^	4.00 (1.00, 10.00) ^†††, ‡‡^
CMAI [points, median (Q1-Q3)]	29.00 (29.00, 29.00)	29.00 (29.00, 29.00)	29.00 (29.00, 32.00) ^†††, ‡‡‡^
MAES [points, median (Q1-Q3)]	5.00 (0.75, 9.00)	5.00 (2.00, 12.00)	14.00 (6.00, 20.50) ^†††, ‡‡^
ADL			
ADL [points, median (Q1-Q3)]	20.00 (20.00, 20.00)	20.00 (20.00, 20.00)	24.00 (20.00, 32.00) ^†††, ‡‡‡^

^†††^*p* < 0.001, AD-D versus NC groups; ^‡‡^*p* < 0.01 and ^‡‡‡^*p* < 0.001, AD-D versus MCI groups; ^*^p < 0.05, ^**^p < 0.01 and ^***^p < 0.001, AD-MCI versus NC groups.

NC, normal control; AD-MCI, mild cognitive impairment due to Alzheimer’s disease; AD-D, dementia due to Alzheimer’s disease; MMSE, Mini-Mental State Examination; MoCA, Montreal Cognitive Assessment; AVLT, Auditory Verbal Learning Test; RFT, Rey-Osterrieth Complex Figure Test; VFT-T, Verbal Fluency Test-all items; BNT, Boston Naming Test; SDMT, Symbol Digit Modalities Test; TMT, Trail Making Test; SCWT, Stroop Color and Word Test; NPI, Neuropsychiatric Inventory; CMAI, Cohen-Mansfield Agitation Inventory; MAES, Modified Apathy Estimate Scale; ADL, Activities of Daily Living.

### 3.4 Associations of eye movement parameters with clinical symptoms of AD

**Figure 3:**
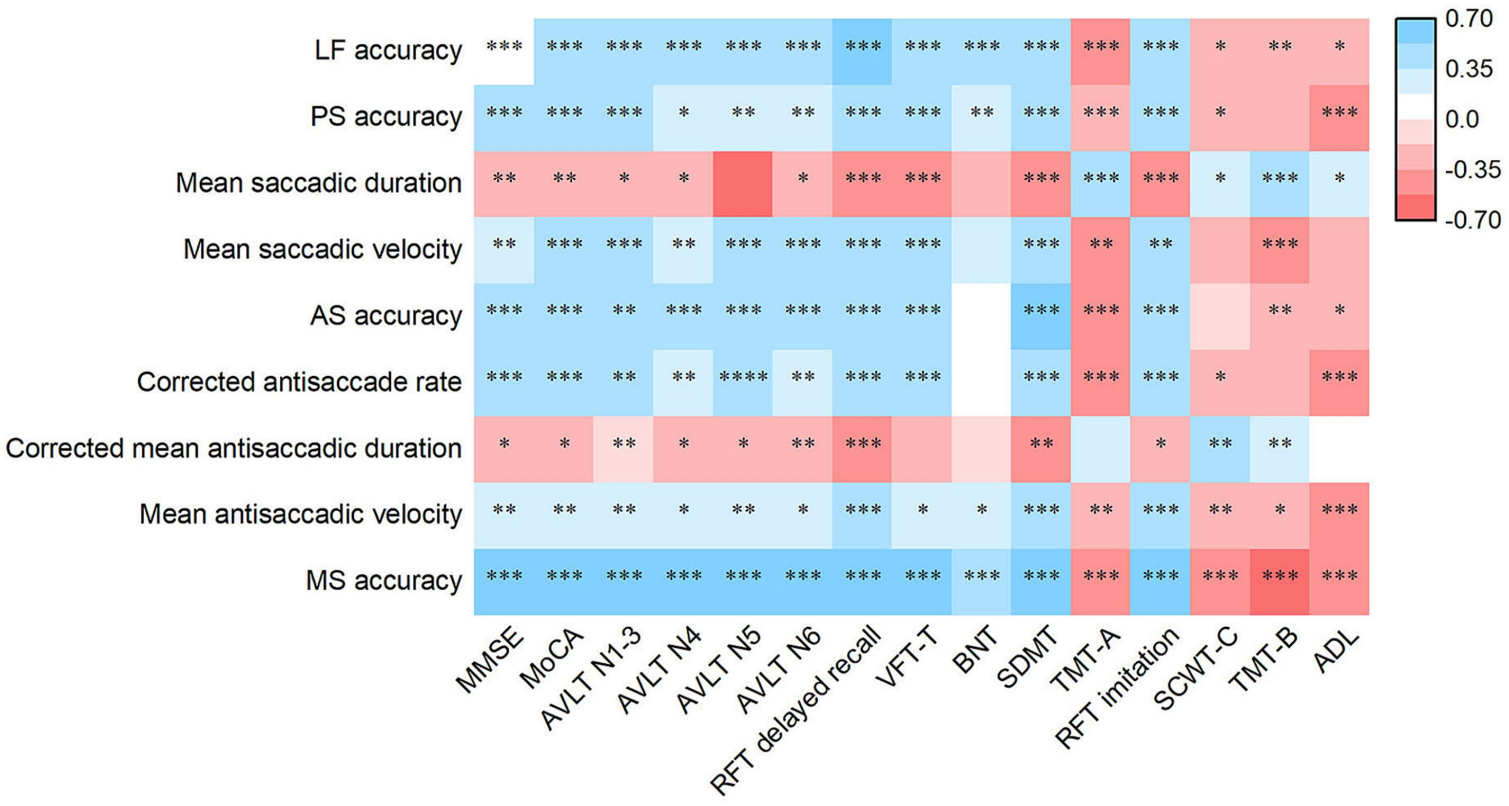
Heatmap of the association of eye movement parameters with the scores of rating scales for clinical symptoms in AD patients. Partial Spearman’s correlation is performed after adjusting for age, sex, duration, years of education, BMI, diastolic blood pressure, *APOE* ε4 status and history of diabetes. ^*^p < 0.05, ^**^p < 0.01 and ^***^p < 0.001. AD, Alzheimer’s disease; *APOE*, Apolipoprotein E; LF, Lateral fixation; PS, prosaccade; AS, antisaccade; MS, memory saccade; MMSE, Mini-Mental State Examination; MoCA, Montreal Cognitive Assessment; AVLT, Auditory Verbal Learning Test; RFT, Rey-Osterrieth Complex Figure Test; VFT-T, Verbal Fluency Test-all items; BNT, Boston Naming Test; SDMT, Symbol Digit Modalities Test; TMT, Trail Making Test; SCWT, Stroop Color and Word Test; ADL, Activities of Daily Living.

For lateral fixation and memory saccades, accuracy was correlated with the scores of all rating scales (all *p* < 0.05; Figure 3). In prosaccades, both accuracy and mean saccadic velocity were positively associated with the MMSE, MoCA, AVLT, RFT delayed recall, VFT-T, SDMT, and RFT imitation scale scores. Accuracy was positively correlated with the BNT score and negatively correlated with the completion time of the TMT-A, SCWT-C, and ADL scales. Moreover, mean saccadic velocity was negatively correlated with TMT-A and TMT-B completion times. The mean saccadic duration was negatively correlated with the MMSE, MoCA, AVLT, RFT delayed recall, VFT-T, SDMT, and RFT imitation scale scores, and positively correlated with the completion times of the TMT-A, SCWT-C, and TMT-B, as well as the ADL score (all p < 0.05). In antisaccades, both accuracy and corrected antisaccade rates demonstrated positive correlations with MMSE, MoCA, AVLT, RFT delayed recall, VFT-T, SDMT, and RFT imitation scale scores, while exhibiting negative correlations with the ADL score. Accuracy was negatively correlated with TMT-A and TMT-B completion times, whereas the corrected antisaccade rate was negatively correlated with TMT-A and SCWT-C completion times. Moreover, the corrected mean antisaccadic duration was negatively correlated with MMSE, MoCA, AVLT, RFT delayed recall, SDMT, and RFT imitation scale scores, and positively correlated with SCWT-C and TMT-B completion times. Finally, the mean antisaccadic velocity was correlated with the scores of all rating scales (all p < 0.05).

**Figure 4:**
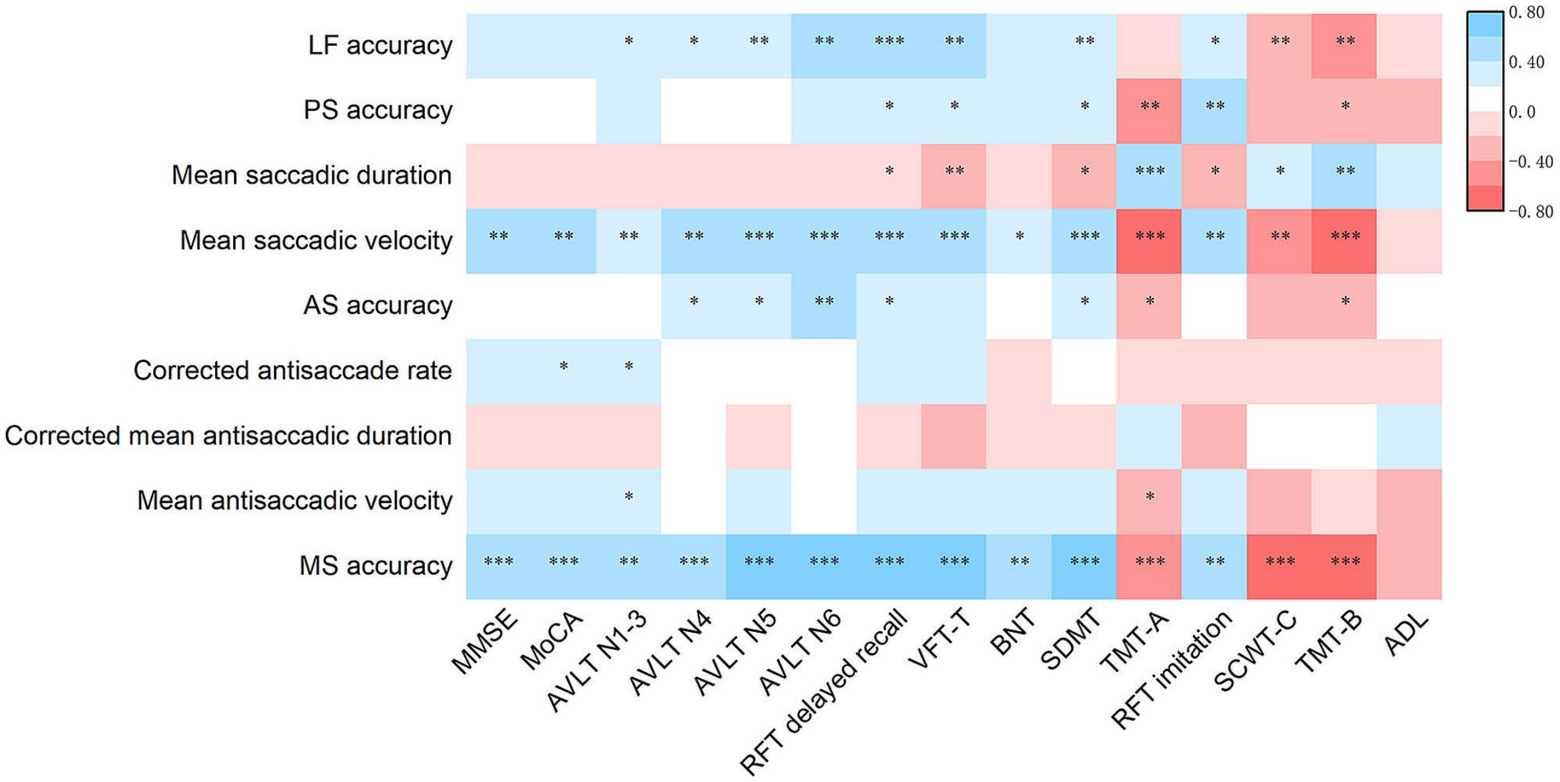
Heatmap of the association of eye movement parameters with the scores of rating scales for clinical symptoms in AD-MCI patients. Partial Spearman’s correlation is performed after adjusting for age, sex, duration, years of education, BMI, diastolic blood pressure, *APOE* ε4 status and history of diabetes. ^*^p < 0.05, ^**^p < 0.01 and ^***^p < 0.001. AD-MCI, mild cognitive impairment due to Alzheimer’s disease; *APOE, apolipoprotein E*; LF, Lateral fixation; PS, prosaccade; AS, antisaccade; MS, memory saccade; MMSE, Mini-Mental State Examination; MoCA, Montreal Cognitive Assessment; AVLT, Auditory Verbal Learning Test; RFT, Rey-Osterrieth Complex Figure Test; VFT-T, Verbal Fluency Test-all items; BNT, Boston Naming Test; SDMT, Symbol Digit Modalities Test; TMT, Trail Making Test; SCWT, Stroop Color and Word Test; ADL, Activities of Daily Living.

**Figure 5:**
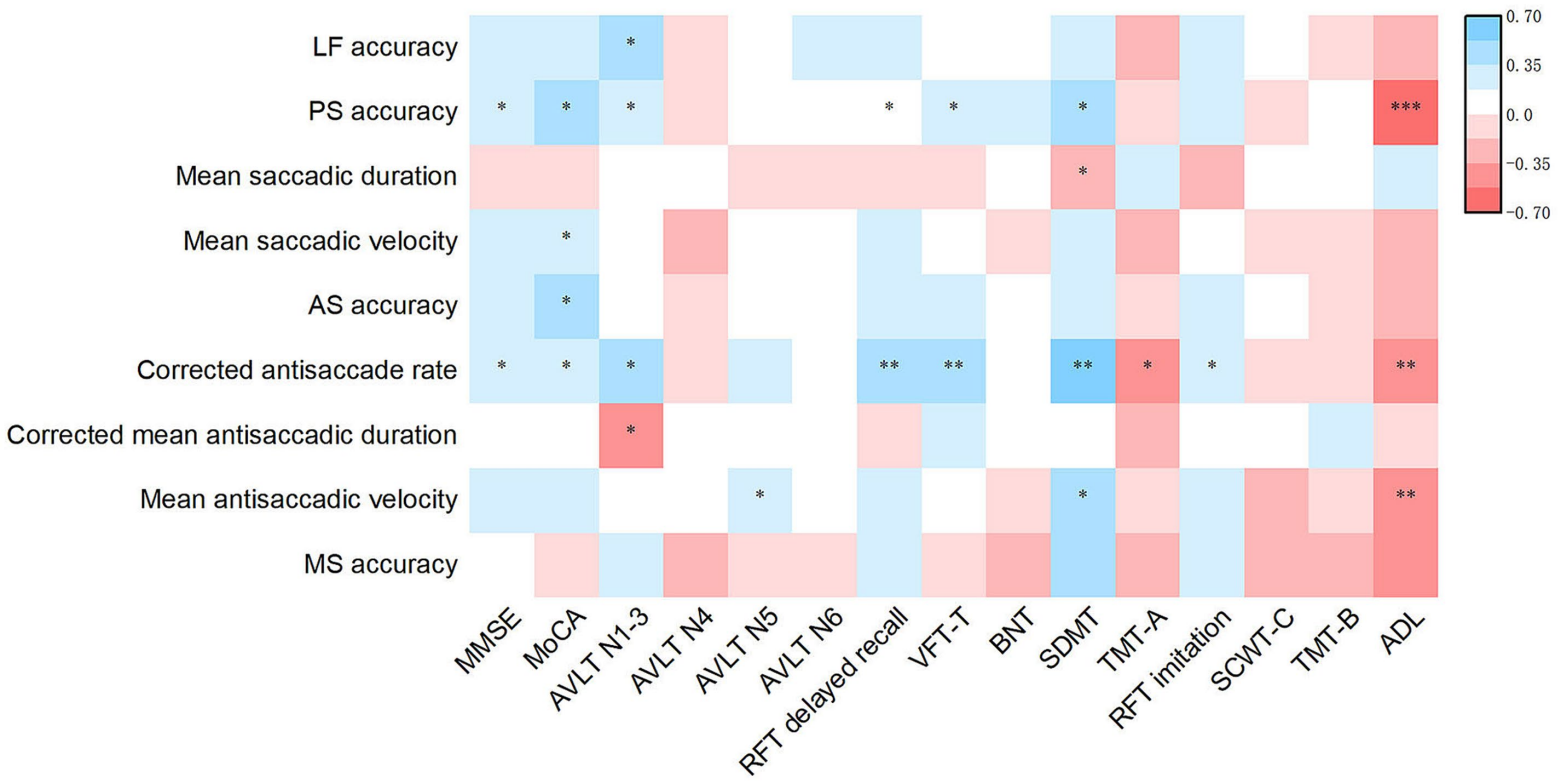
Heatmap of the association of eye movement parameters with the scores of rating scales for clinical symptoms in AD-D patients. Partial Spearman’s correlation is performed after adjusting for age, sex, duration, years of education, BMI, diastolic blood pressure, *APOE* ε4 status and history of diabetes. ^*^p < 0.05, ^**^p < 0.01 and ^***^p < 0.001. AD-D, dementia due to Alzheimer’s disease; *APOE*, Apolipoprotein E; LF, Lateral fixation; PS, prosaccade; AS, antisaccade; MS, memory saccade; MMSE, Mini-Mental State Examination; MoCA, Montreal Cognitive Assessment; AVLT, Auditory Verbal Learning Test; RFT, Rey-Osterrieth Complex Figure Test; VFT-T, Verbal Fluency Test-all items; BNT, Boston Naming Test; SDMT, Symbol Digit Modalities Test; TMT, Trail Making Test; SCWT, Stroop Color and Word Test; ADL, Activities of Daily Living.

In the AD-MCI group, the accuracy of lateral fixation, prosaccades, antisaccades, memory saccades, and mean saccadic duration and velocity remained significantly correlated with the performances on the TMT-A, TMT-B, SDMT, RFT delayed recall, and RFT imitation scales. Conversely, the correlations of eye movement performance with MMSE, MoCA, and ADL scale scores diminished significantly. However, the corrected antisaccade rate, corrected mean antisaccadic duration, and mean antisaccadic velocity exhibited weak correlations with the scores on the above rating scales (Figure 4). In the AD-D group, most correlations between eye movement parameters and the scores on the above rating scales disappeared. The correlations of prosaccade accuracy and corrected antisaccade rate with MMSE, MoCA, AVLT N1-3, RFT delayed recall, VFT-T, SDMT, and ADL scores remained significant (Figure 5).

Among patients with AD, 79 cases (70.5%) were identified as having diabetes, while 33 (29.5%) did not exhibit this comorbidity. In patients without diabetes, the correlations of eye movement parameters with cognitive function, and ADL scale scores remained significant, consistent with the findings observed in all patients with AD. Conversely, in patients with AD and diabetes, the correlations of mean saccadic velocity, antisaccade accuracy, and memory saccade accuracy with the scores of the rating scales for clinical symptoms were similar to those observed in all AD patients (Supplementary Figures S1, S2). Other indicators were exclusively associated with visuospatial, attentional, and executive functions in patients with AD and diabetes.

### 3.5 The association between eye movement parameters and AD

**Figure 6:**
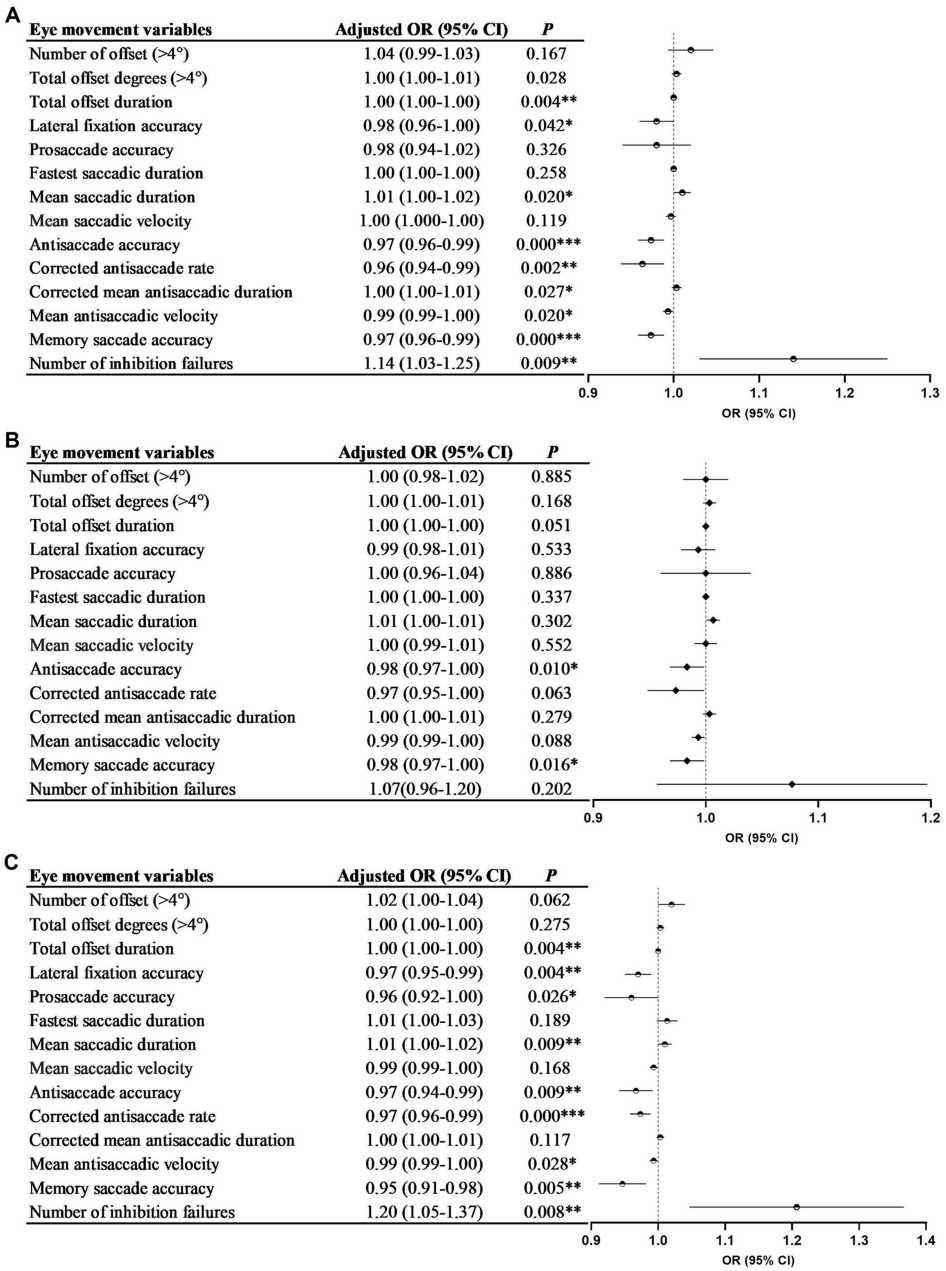
The association between eye movement parameters and AD (A), AD-MCI (B), and AD-D (C). Figures 2A,B were adjusted for age, sex, years of education, BMI, diastolic blood pressure and history of diabetes; Figure 2C was adjusted for age, sex, duration, years of education, BMI, diastolic blood pressure, carrying of *APOE* ε4 allele and history of diabetes. AD, Alzheimer’s disease; AD-MCI, mild cognitive impairment due to Alzheimer’s disease; AD-D, dementia due to Alzheimer’s disease; BMI, body mass index; *APOE*, apolipoprotein E; OR, odds ratio. ^*^p < 0.05, ^**^p < 0.01 and ^***^p < 0.001.

After adjusting for confounding factors, lateral fixation accuracy, antisaccade accuracy, corrected antisaccade rate, mean antisaccadic velocity, and memory saccade accuracy were found to be negatively associated with AD. Conversely, the mean saccadic duration and number of inhibition failures were positively associated with AD. The accuracy of the antisaccades and memory saccades was negatively associated with AD-MCI. The accuracy of lateral fixation, prosaccades, and antisaccades, corrected antisaccade rate, mean antisaccadic velocity, and memory saccade accuracy were negatively associated with AD-D. In contrast, the mean saccadic duration and number of inhibition failures were positively associated with AD-D (all *p* < 0.05; Figures 6A–C).

### 3.6 Predictive value of eye movement parameters for AD

**Figure 7:**
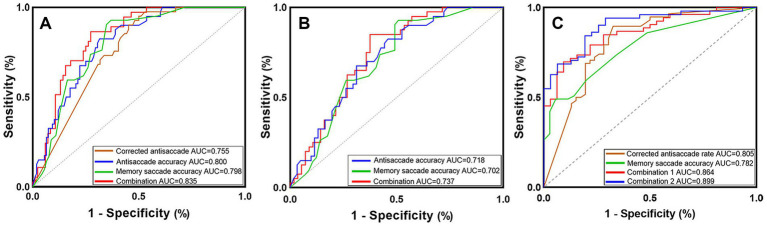
ROCs for discriminating AD (A), AD-MCI (B), and AD-D (C) patients. (A) Eye movement parameters and the combination of lateral fixation accuracy, mean saccadic duration, antisaccade accuracy, corrected antisaccade rate, mean antisaccadic velocity, memory saccade accuracy, and the number of inhibition failures predicted the progression from NC to AD; (B) The accuracy of antisaccade and memory saccade and their combination predicted the progression from NC to AD-MCI; (C) Combination 1 included lateral fixation accuracy, mean saccadic duration, antisaccade accuracy, corrected antisaccade rate, mean antisaccadic velocity, memory saccade accuracy, and the number of inhibition failures. Combination 2 further combined age, years of education, and carrying of *APOE* ε4 allele based on Combination 1 to predict the progression from AD-MCI to AD-D. ROC, receiver operator characteristic; AD, Alzheimer’s disease; AD-MCI, mild cognitive impairment due to Alzheimer’s disease; AD-D, dementia due to Alzheimer’s disease; NC, normal control; *APOE*, apolipoprotein E; AUC, area under the curve.

The AUC for the antisaccade accuracy was 0.800, with a sensitivity, specificity, and accuracy of 68.5, 82.5, and 72.3%, respectively. The AUC for memory saccade accuracy was 0.798, with a sensitivity, specificity, and accuracy of 63.0, 92.9, and 72.4%, respectively. These metrics indicated that the accuracy of antisaccade and memory saccade was more effective in differentiating patients with AD from NC than prosaccade and lateral fixation metrics. The combination of lateral fixation accuracy, mean saccadic duration, antisaccade accuracy, corrected antisaccade rate, mean antisaccadic velocity, memory saccade accuracy, and the number of inhibition failures were the most effective in predicting the progression from NC to AD, exhibiting an AUC of 0.835, along with a sensitivity, specificity, and accuracy of 72.6, 86.5, and 76.9%, respectively (Figure 7A; Supplementary Table S1).

The combination of the accuracy of antisaccades and memory saccades represented the most effective variables for predicting the progression from NC to AD-MCI, yielding an AUC of 0.737 with a sensitivity, specificity, and accuracy of 62.5, 85.0, and 71.9%, respectively (Figure 7B; Supplementary Table S2).

The corrected antisaccade rate exhibited greater efficacy in differentiating patients with AD-MCI from those with AD-D, with an AUC of 0.805 and sensitivity, specificity, and accuracy of 67.4, 89.7, and 79.8%, respectively. The combination of lateral fixation accuracy, mean saccadic duration, antisaccade accuracy, corrected antisaccade rate, mean antisaccadic velocity, memory saccade accuracy, and number of inhibition failures displayed an AUC of 0.864, with a sensitivity, specificity, and accuracy of 90.6, 69.8, and 77.6%, respectively, in predicting the progression from AD-MCI to AD-D. Furthermore, when combined with age, years of education, and *APOE* ε4 allele status, the AUC was increased to 0.899, with sensitivity, specificity, and accuracy of 71.0, 94.1, and 85.4%, respectively (Figure 7C; Supplementary Table S3).

## 4 Discussion

The characteristics of eye fixation and saccadic movement in patients with AD were examined, their relationship with the clinical symptoms of AD was explored, and the predictive value of eye movement parameters for the diagnosis and progression of AD was evaluated in this study.

The degree and duration of offset of lateral fixation were compared to assess fixation stability in patients with AD and the AD-D group exhibited more frequent offsets (>4°), greater total offset degrees (>4°), and lower accuracy than the NC and AD-MCI groups. However, no significant differences were observed between the NC and AD-MCI groups. Additionally, the AD-D group exhibited the longest total offset duration, followed by the AD-MCI and NC groups (Table 2), suggesting that fixation instability in at the early stage worsened with the progression of AD; thus, the total offset duration might potentially serve as an indicator for the early identification and progression monitoring of patients with AD. Compared to other neurodegenerative diseases, AD and related disorders are characterized by fixation instability (Sekar et al., 2024). Patients with AD reportedly exhibited more unstable fixations than cognitively normal individuals, suggesting that fixation could serve as a useful tool for identifying patients with AD (Ciudin et al., 2024). The brain regions implicated in fixation include the occipital eye field, middle temporal area, medial temporal area, DLPFC, FEF, and the brainstem (Zee, 2021). Damage to the functional or structural integrity of these regions along with impairments in attention and visuospatial functions may contribute to fixation instability (Martinez-Conde et al., 2013).

In this study, the AD-D group presented a lower accuracy and longer saccadic duration than the NC and AD-MCI groups and a lower mean saccadic velocity than the NC group (Table 2). To initiate saccades, excitatory activities originating from the FEF, PEF, and supplementary eye field (SEF), along with inhibitory signals from the substantia nigra, are sent to the superior colliculus and then projected to the saccade burst generator in the reticular formation, which send instructions to the ocular motor neurons for eye movement (Wu et al., 2020). Thus, the performance observed in patients with AD suggests a dysfunction of these associated cortical and subcortical regions. The decreased accuracy might be attributed to impaired disengagement and reorientation induced by AD (Perry and Hodges, 1999; Parasuraman et al., 1992). The assessment of fastest and mean saccadic duration in the present study revealed that patients with AD-D required significantly more time to accurately execute prosaccades. This phenomenon may be associated with the damage sustained by the brain structures and pathways necessary for executing prosaccades, which is exacerbated by AD pathology. Additionally, it may be influenced by attention disorders (Clark et al., 2015) and extended processing times for movement and sensation within the brain (Chehrehnegar et al., 2019). However, no significant differences were observed in prosaccade latency between patients with AD and NC, which is consistent with findings from prior studies. Patients with AD might exhibit normal prosaccades, demonstrating comparable latency (Mosimann et al., 2004; Crawford et al., 2005). Prosaccade latency could reportedly differentiate patients with AD-D from NC, but did not distinguish those with MCI from NC. Considering that saccadic disorders are indicative of a decline in neurological and cognitive functions, these inconsistent findings may be explained by the variability in disease severity across different studies (Molitor et al., 2015). Future research employing longitudinal follow-up is crucial for a thorough investigation and analysis of these findings. Visuomotor pathways associated with saccadic velocity are primarily located in the brainstem (Zhou et al., 2015). By contrast, the brain regions affected in patients with AD-MCI primarily reside in the cortex, thereby rendering their saccadic velocity largely unaffected.

The order of antisaccade accuracy in this study ranged from low to high in the AD-D, AD-MCI, and NC groups, suggesting that accuracy contributed to the early identification of AD. Compared to the NC and AD-MCI groups, the AD-D group exhibited a lower corrected antisaccade rate and longer corrected mean antisaccadic duration (Table 2), which is consistent with previous findings (Opwonya et al., 2022b; Chehrehnegar et al., 2022; Crawford et al., 2013; Wilcockson et al., 2019; Koçoğlu et al., 2021). The antisaccade process entails the activation of regions, including the DLPFC, FEF, and basal ganglia, to suppress reflexive saccades toward the target. Subsequently, cortical areas, such as the parietal cortex, FEF, SEF, and basal ganglia direct the saccade away from the target. FEF initiates antisaccades through the saccadic system (Coe and Munoz, 2017). During this process, the posterior region of the anterior cingulate gyrus is involved in controlling intentional saccades. The “cingulate eye field” located between Brodmann areas 23 and 24, prepares all involved frontal ocular motor areas through an intentional motivational process to act in forthcoming movement behaviors. The DLPFC is regulated by the cingulate eye field (Gaymard et al., 1998). At the early stages of AD, inhibitory control and working memory, primarily mediated by the DLPFC, as well as the brain regions responsible for initiating correct antisaccades, including the FEF, PEF, and SEF, are often impaired, potentially leading to decreased antisaccade accuracy. Patients with AD often exhibit a reduced tendency to correct errors, which could be attributed to alterations in self-monitoring and error correction networks involving the frontal and anterior cingulate cortices (Wilcockson et al., 2019). Limited investigations have been conducted on antisaccadic velocity, with only a few results available for patients with AD-MCI. In our study, the AD-D group exhibited a lower mean antisaccadic velocity than the NC group; however, no significant differences were observed between the AD-MCI group and the other groups. This indicates that the mean velocity of both the antisaccades and prosaccades remained unchanged during the early stages of AD. The AD-D group exhibited a longer latency and mean antisaccadic duration than the AD-MCI group, although these differences did not reach statistical significance (Table 2). To date, the results regarding antisaccade latency between patients with AD and NC have been inconsistent (Kahana Levy et al., 2018; Garbutt et al., 2008), highlighting the need for further investigation and a larger sample size to validate these findings.

In the present study, the order of memory saccade accuracy from low to high was as follows: AD-D, AD-MCI, and NC groups, indicating its potential as an early indicator of AD. Additionally, compared to the NC and AD-MCI groups, the AD-D group exhibited a significantly greater incidence of inhibition failures (Table 2). The main cortical region associated with memory saccades is the DLPFC, although other cortical eye fields are also involved (Kastner et al., 2007). A functional magnetic resonance study conducted in healthy adults during memory saccades demonstrated significant activation of the FEF, SEF, parietal cortex, posterior inferior frontal gyrus, posterior superior temporal gyrus, and posterior dorsal anterior cingulate cortex (Ozyurt et al., 2006). Memory saccades constitute a form of short-term visuospatial working memory. During the brief initial phase of visual–spatial integration and subsequent stimulus presentation, the posterior parietal cortex is involved in regulating saccade accuracy. In the memory phase, the DLPFC and FEF integrate the incoming visual information and encode and store the target location. Upon the appearance of the saccade command, the DLPFC extracts the stored information and simultaneously issues a command to the saccade pathway, prompting the eyes to saccade toward the target. FEF is primarily involved in the initiation of memory saccades (Pierrot-Deseilligny et al., 2002). Degeneration of brain regions related to memory saccades, particularly the DLPFC in patients with AD, coupled with reduced fiber connectivity between the DLPFC and various cortical regions, results in the impairment of visuospatial working memory (Liang et al., 2011). This degeneration leads to abnormalities across various stages of visual and/or spatial information processing, storage, retention, and extraction, resulting in lower accuracy and more inhibition failures. Patients with AD reportedly exhibited longer memory saccade latency than NC (Lage et al., 2020). In contrast, our findings revealed a trend indicating shorter latency in patients with AD compared to NC, potentially attributable to the failure of preemptive inhibitory processes to suppress reflexive motor output from the superior colliculus (Everling et al., 1998), thereby indicating impaired inhibitory control in patients with AD-D. Upon the appearance of the target dots, participants were unable to inhibit the reflexive saccade toward these stimuli, leading to express latency saccades.

In the present study, lower accuracy in lateral fixation was associated with significantly impaired functions of global cognition and individual cognitive domains, and poorer ADL in patients with AD. In prosaccades, lower accuracy, longer mean saccadic duration, and lower mean saccadic velocity were indicative of poorer performance in the global cognition and individual cognitive domains. Lower accuracy was associated with more significant impairments in ADL. In antisaccades, lower accuracy, corrected antisaccade rate, and lower mean antisaccadic velocity were linked to poorer performance in global cognition, individual cognitive domains, and ADL. Extended corrected mean antisaccadic duration was associated with worse global cognition and individual cognitive domains, except language. Decreased accuracy in memory saccades was indicative of impaired global cognition, other cognitive domains, and ADL (Figure 3). Notably, changes in eye movement parameters exhibited the strongest association with attention, visual memory, and visuospatial ability, suggesting that eye movement metrics accurately reflect the clinical symptoms of patients with AD. On the one hand, the brain regions governing eye movement overlap with those related to various cognitive domains in patients with AD; on the other hand, multiple cognitive impairments observed in AD can affect different aspects of eye movements. No significant associations were observed between eye movements and neuropsychiatric symptoms.

Further analysis indicated that eye movement parameters in patients with AD-MCI, including accuracy of lateral fixation, prosaccades, antisaccades, and memory saccades, along with mean saccadic duration and velocity, were most closely associated with attention and executive function. Memory (verbal and visual) and visuospatial functions exhibited some relevance; however, they exhibited no correlation with global cognition or ADL (Figure 4). Executive function was reportedly correlated with prosaccade accuracy; however, no such correlation existed with accuracy or corrected rates of antisaccades in patients with MCI (Opwonya et al., 2022b). Patients with early AD may manifest impaired attention, execution, and visuospatial ability governed by the prefrontal and parietal lobes, leading to abnormal eye movements. Consequently, eye movements may serve as early indicators of compromised function in these cognitive domains.

In patients with AD-D, most eye movement parameters did not demonstrate significant correlations with cognitive function; however, certain indicators remained associated with global cognition, memory, attention, and ADL. Specifically, lower prosaccade accuracy and corrected rate of antisaccades were associated with more severely compromised global cognition, memory, attention, and ADL (Figure 5). In patients with AD-D, extensive neurodegeneration occurs across multiple brain regions, including those implicated in eye movements, potentially leading to severe impairment in these movements and diminishing their relationship with cognitive function. Additionally, patients with AD-D experience a more pronounced cognitive decline, rendering eye movement parameters ineffective indicators of cognitive status.

Our results suggest that diabetes modifies the relationship between eye movement performance and clinical symptoms in patients with AD. In patients without diabetes, the associations between eye movement performance and global cognition, all cognitive domains, and ADL were comparable to those observed in all patients with AD (Supplementary Figure S1). In patients with diabetes, many of these correlations disappeared (Supplementary Figure S2). However, eye movements continued to exhibit associations with visuospatial ability, attention, and executive function. This discrepancy may be attributed to several factors. First, diabetes adversely affects the oculomotor nerve and white matter fibers, potentially decreasing the coordination and flexibility of eye movements (D'Addio et al., 2022). Second, diabetes accelerates neurodegeneration within the brain (Santiago et al., 2023), thereby exacerbating cognitive decline and diminishing the association between cognitive function and eye movement performance. Finally, the limited sample size of patients with diabetes in the current study may have impacted the statistical power, necessitating careful interpretation of these results.

In this study, the accuracy of antisaccades and memory saccades demonstrated enhanced predictive value for AD and AD-MCI. The combination of antisaccade and memory saccade accuracy exhibited the highest predictive power for AD-MCI (AUC = 0.737), indicating that these parameters, as well as their combinations, emerge at the early stage of AD and hold promise as potential markers for early diagnosis (Figures 7A,B; Supplementary Tables S1, S2). The combination of eye movement parameters demonstrated the most robust ability to predict the progression from AD-MCI to AD-D (AUC = 0.864), which was further enhanced when integrated with age, education level, and *APOE* ε4 allele status (AUC = 0.899, Figure 7C; Supplementary Table S3). These data suggest that antisaccades and memory saccades are more sensitive in identifying early AD, as they rely on advanced levels of cognitive and executive processing (Kirova et al., 2015).

This study demonstrates several significant strengths. First, this study provided the first comprehensive description of fixation stability by using lateral fixation tests in patients with AD at different stages. Second, this investigation also conducted memory saccades to evaluate the characteristics of voluntary saccade in patients with AD at different stages, in addition to antisaccade. Additionally, this study conducted a comprehensive assessment of clinical symptoms of AD, including cognitive function, neuropsychiatric symptoms, and ADL, analyzed their associations with eye movement parameters, and elucidated the effects of disease severity and diabetes on these associations. Finally, the current results provided the first evidence supporting the feasibility of utilizing memory saccade and a combination of multiple saccadic movements alongside lateral fixation to identify AD and predict its progression.

This study has several inherent limitations. Given that this was a cross-sectional study, it is imperative to conduct longitudinal investigations to gain a deeper understanding of the dynamic changes in eye movements. Further validation is necessary, particularly with a larger sample of patients with AD, including those diagnosed with diabetes. Although standardized and comprehensive instructions were provided along with pretests, participants may not consistently adhere to the intricate instructions, which could potentially undermine data validity and complicate the interpretation of the results. The incorporation of additional methods, such as visual cues and step-by-step guidance, is essential to assist participants in better understanding the instructions. Recruiting patients with AD at an earlier stage and integrating eye movements with naturalistic scenes are also necessary. This investigation focused on gaze deviations attributable to attention deficits, thereby neglecting physiological fixational instability, such as microsaccades, which could have influenced our results (Egaña et al., 2013).

In summary, patients with AD exhibit a variety of abnormal eye movements, including not only the extensively studied prosaccades and antisaccades but also lateral fixation and memory saccades. Some indicators exhibit abnormalities at the MCI stage, which tend to exacerbate with disease progression. Abnormal eye movements are related to clinical symptoms, and these associations are influenced by AD severity. Patients demonstrate associations between eye movement parameters and specific cognitive domains, particularly attention and executive function at the MCI stage, whereas these parameters show a closer association with global cognition and ADL at the dementia stage. These findings establish a theoretical foundation for using eye movement parameters to identify cognitive impairment and underscore the necessity for early eye movement tests, especially memory saccades, to facilitate the screening of cognitive impairment. This study demonstrates the feasibility of memory saccades, along with antisaccades, for identifying AD and assessing disease progression. Combining multiple eye movement paradigms, including prosaccades and lateral fixation, optimizes the predictive results. This study suggests that eye movement parameters have the potential to serve as novel biomarkers for early screening, symptom assessment, and progression monitoring for patients with AD.

## Data Availability

The raw data supporting the conclusions of this article will be made available by the authors, without undue reservation.
