# Supplementary material for: Abnormal eye movements: relationship with clinical symptoms and predictive value for Alzheimer’s disease
Source: Front Aging Neurosci. 2024 Nov 21;16:1471698. doi: 10.3389/fnagi.2024.1471698 (PMC11617582; doi:10.3389/fnagi.2024.1471698)
Supplement: Supplementary file 1 [file Table_1.docx]

Supplementary Material

# Supplementary Figures

**
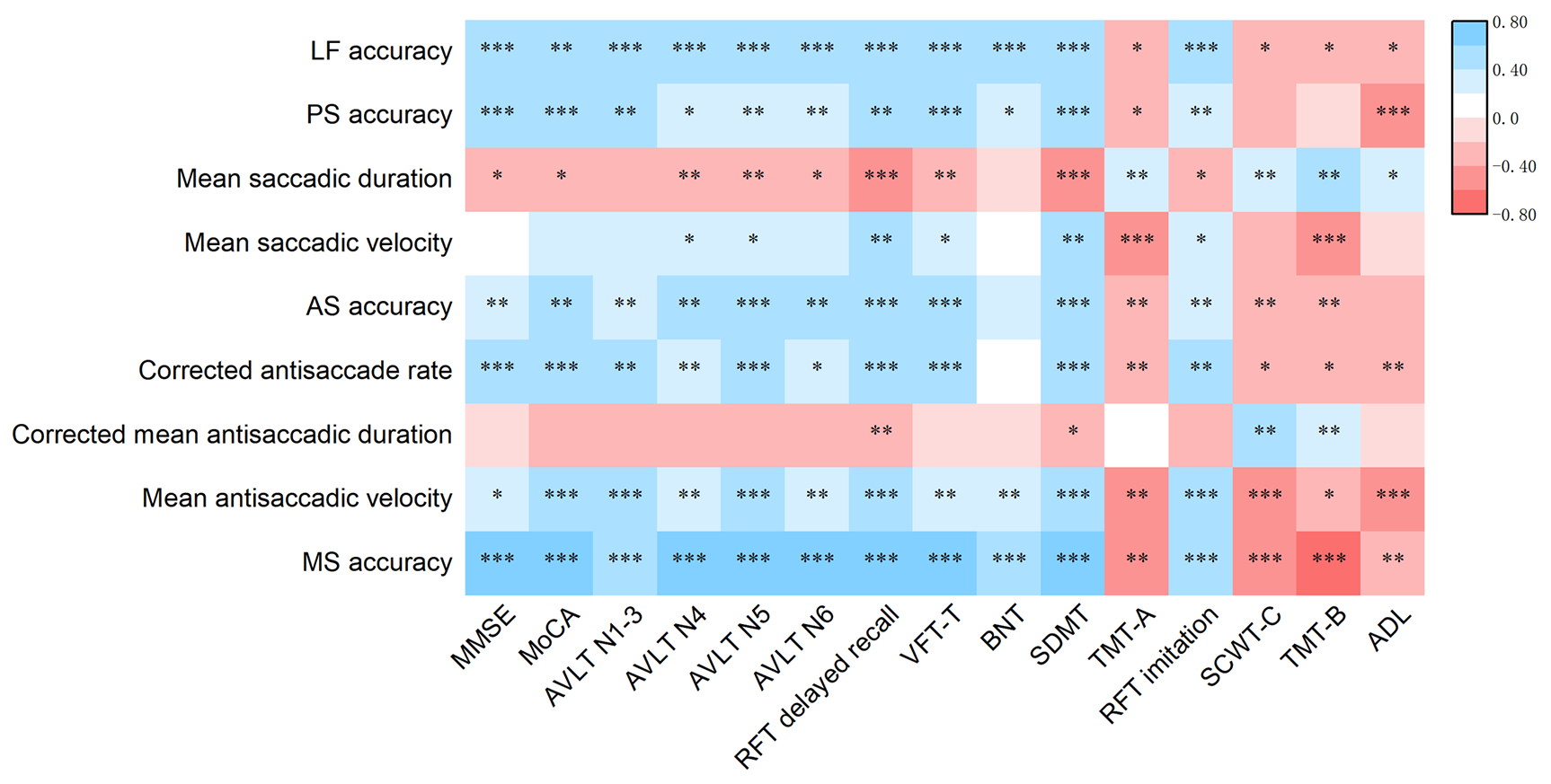
**

**Figure S1 Heatmap of the association of eye movement parameters with the scores of rating scales for clinical symptoms in AD patients without diabetes (n = 79).** Partial Spearman’s correlation is performed after adjusting for age, sex, duration, years of education, BMI, diastolic blood pressure and *APOE* ε4 status. ^*^ P < 0.05, ^**^ P < 0.01 and ^***^ P < 0.001. Abbreviation: AD, Alzheimer’s disease; *APOE, apolipoprotein E*; LF, lateral fixation; PS, prosaccade; AS, antisaccade; MS, memory saccade; MMSE, Mini-Mental State Examination; MoCA, Montreal Cognitive Assessment; AVLT, Auditory Verbal Learning Test; RFT, Rey-Osterrieth Complex Figure Test; VFT-T, Verbal Fluency Test-all items; BNT, Boston Naming Test; SDMT, Symbol Digit Modalities Test; TMT, Trail Making Test; SCWT, Stroop Color and Word Test; ADL, Activities of Daily Living.


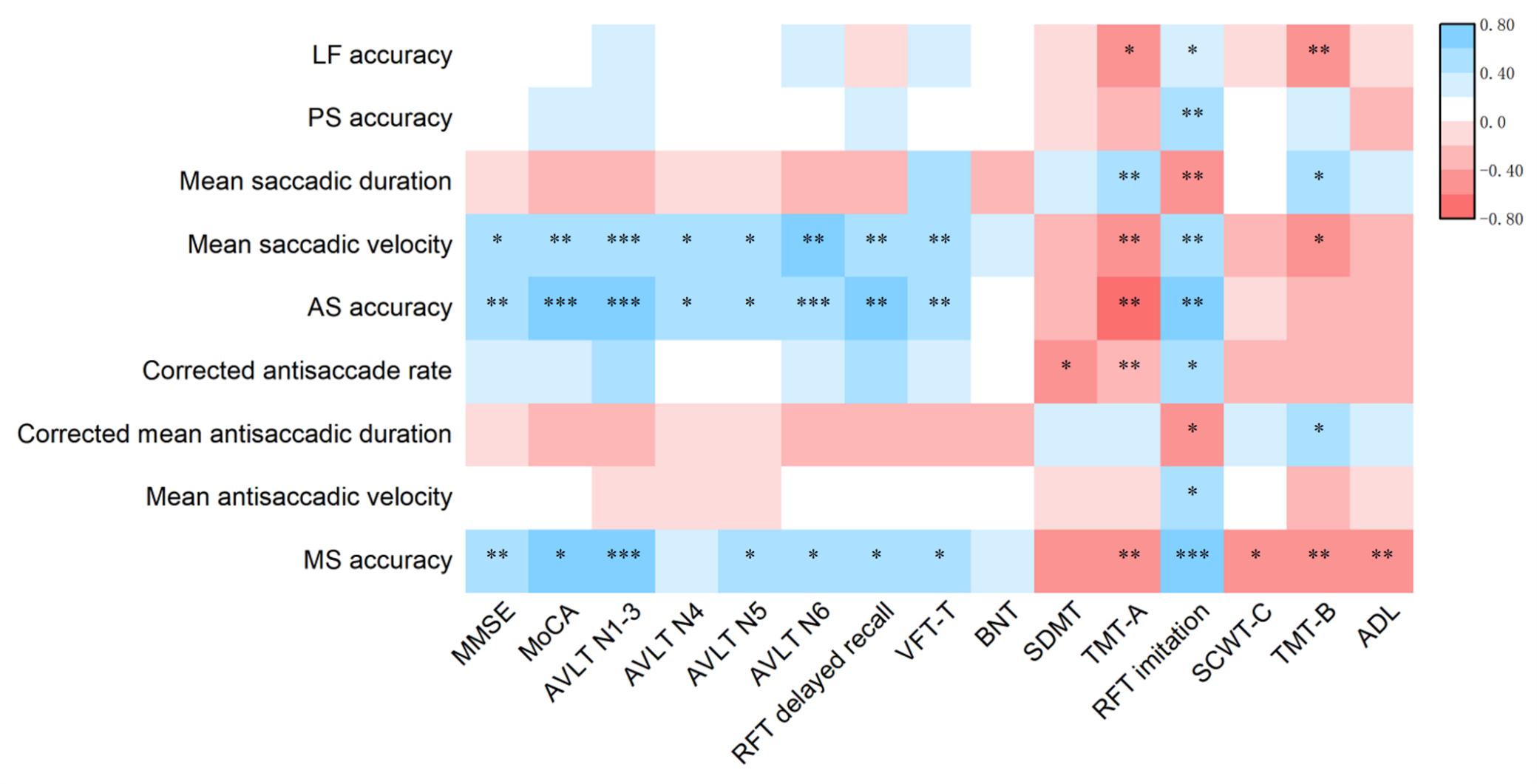


**Figure S2 Heatmap of the association of eye movement parameters with the scores of rating scales for clinical symptoms in AD patients with diabetes (n = 33).** Partial Spearman’s correlation is performed after adjusting for age, sex, duration, years of education, BMI, diastolic blood pressure, and *APOE* ε4 status. ^*^ P < 0.05, ^**^ P < 0.01 and ^***^ P < 0.001. Abbreviation: AD, Alzheimer’s disease; *APOE, apolipoprotein E*; LF, Lateral fixation; PS, prosaccade; AS, antisaccade; MS, memory saccade; MMSE, Mini-Mental State Examination; MoCA, Montreal Cognitive Assessment; AVLT, Auditory Verbal Learning Test; RFT, Rey-Osterrieth Complex Figure Test; VFT-T, Verbal Fluency Test-all items; BNT, Boston Naming Test; SDMT, Symbol Digit Modalities Test; TMT, Trail Making Test; SCWT, Stroop Color and Word Test; ADL, Activities of Daily Living.

# Supplementary Tables

**Table S1** ROC analyses of different eye movement parameters and their combination to predict the progression from NC to AD

| Parameters | AUC | 95% CI | Sensitivity | Specificity | Accuracy |
| --- | --- | --- | --- | --- | --- |
| Lateral fixation accuracy | 0.696^***^ | 0.598~0.793 | 59.8% | 75.0% | 63.8% |
| Mean saccadic duration | 0.647^**^ | 0.557~0.738 | 49.1% | 80.5% | 57.8% |
| Antisaccade accuracy | 0.800^***^ | 0.728~0.871 | 68.5% | 82.5% | 72.3% |
| Corrected antisaccade rate | 0.755^***^ | 0.678~0.832 | 50.0% | 95.1% | 62.8% |
| Mean antisaccadic velocity | 0.639^**^ | 0.544~0.734 | 41.5% | 75.2% | 53.4% |
| Memory saccade accuracy | 0.798^***^ | 0.723~0.873 | 63.0% | 92.9% | 72.4% |
| Number of inhibition failures | 0.660^**^ | 0.562~0.758 | 42.2% | 85.7% | 56.1% |
| Combination | 0.835^***^ | 0.764~0.905 | 72.6% | 86.5% | 76.9% |

^**^ P < 0.01 and ^***^ P < 0.001. Abbreviation: ROC, receiver operator characteristic; NC, normal control; AD, Alzheimer’s disease; AUC, area under the curve; CI, confidence interval.

**Table S2** ROC analyses of antisaccade accuracy, memory saccade accuracy and their combination to predict the progression from NC to AD-MCI

| Parameters | AUC | 95% CI | Sensitivity | Specificity | Accuracy |
| --- | --- | --- | --- | --- | --- |
| Antisaccade accuracy | 0.718^***^ | 0.619~0.816 | 54.1% | 82.5% | 63.2% |
| Memory saccade accuracy | 0.702^***^ | 0.600~0.805 | 49.1% | 92.9% | 67.7% |
| Combination | 0.737^***^ | 0.639~0.835 | 62.5% | 85.0% | 71.9% |

^***^ P < 0.001. Abbreviation: ROC, receiver operator characteristic; NC, normal control; AD-MCI, mild cognitive impairment due to Alzheimer's disease; AUC, area under the curve; CI, confidence interval.

**Table S3** ROC analyses of different eye movement variables and their combination to predict the progression from AD-MCI to AD-D

| Parameters | AUC | 95% CI | Sensitivity | Specificity | Accuracy |
| --- | --- | --- | --- | --- | --- |
| Lateral fixation accuracy | 0.747^***^ | 0.656~0.838 | 85.7% | 57.1% | 69.6% |
| Mean saccadic duration | 0.737^***^ | 0.642~0.832 | 65.9% | 75.8% | 71.7% |
| Antisaccade accuracy | 0.708^***^ | 0.611~0.805 | 74.5% | 62.3% | 67.6% |
| Corrected antisaccade rate | 0.805^***^ | 0.716~0.894 | 67.4% | 89.7% | 79.8% |
| Memory saccade accuracy | 0.782^***^ | 0.690~0.875 | 94.3% | 49.1% | 66.3% |
| Number of inhibition failures | 0.664^**^ | 0.545~0.784 | 57.1% | 76.4% | 68.9% |
| Combination 1 | 0.864^***^ | 0.788~0.939 | 90.6% | 69.8% | 77.6% |
| Combination 2 | 0.899^***^ | 0.833~0.966 | 71.0% | 94.1% | 85.4% |

^**^ P < 0.01 and ^***^ P < 0.001. Combination 1 included all eye movement variables in the table. Combination 2 added the *APOE* ε4 allele, age, and years of education to combination 1. Abbreviation: ROC, receiver operator characteristic; AD-MCI, mild cognitive impairment due to Alzheimer's disease; AD-D, dementia due to Alzheimer’s disease; AUC, area under the curve; CI, confidence interval; *APOE, apolipoprotein E*.
